# (1*S*,2*R*,3*R*,8*R*,10*S*)-3-Chloro-2,8-dihy­droxy-3,7-dimethyl-11-methyl­idene-13-oxabicyclo­[8.3.0]tridec-6-en-12-one

**DOI:** 10.1107/S1600536811039717

**Published:** 2011-10-05

**Authors:** Mohamed Moumou, Ahmed Benharref, Jean-Claude Daran, Ahmed Elhakmaoui, Mohamed Akssira, Moha Berraho

**Affiliations:** a Laboratoire de Chimie Biomoleculaire, Substances Naturelles et Réactivité, URAC16, Faculté des Sciences Semlalia, BP 2390 Bd My Abdellah, 40000 Marrakech, Morocco; b Laboratoire de Chimie de Coordination, 205 route de Narbonne, 31077 Toulouse Cedex 04, France; c Laboratoire de Chimie Bioorganique et Analytique, URAC 22. BP 146, FSTM, Université Hassan II, Mohammedia–Casablanca 20810 Mohammedia, Morocco

## Abstract

The title compound, C_15_H_21_ClO_4_, was synthesized from 9α-hy­droxy­parthenolide (9α-hy­droxy-4,8-dimethyl-12-methylen-3,14-dioxatricyclo­[9.3.0.0^2,4^]tetra­dec-7-en-13-one), which was isolated from the chloro­form extract of the aerial parts of *Anvillea radiata*. The mol­ecule is built up from fused five- and ten-membered rings. The five-membered lactone ring has an envelope conformation with the flap atom, C(H)-C-C(H), displaced by 0.2325 (15) Å from the mean plane through the remaining four atoms, whereas the ten-membered ring displays an approximate chair–chair conformation. The dihedral angle between the two rings is 66.4 (2)°. In the crystal, mol­ecules are linked into chains propagating along the *a* axis by O—H⋯O hydrogen bonds.

## Related literature

For the isolation and biological activity of 9α-hy­droxy­parthenolide, see: El Hassany *et al.* (2004 ▶). For the reactivity of this sesquiterpene, see: Castaneda-Acosta *et al.* (1993 ▶); Neukirch *et al.* (2003 ▶); Hwang *et al.* (2006 ▶); Neelakantan *et al.* (2009 ▶). For conformational analysis, see: Cremer & Pople (1975 ▶)
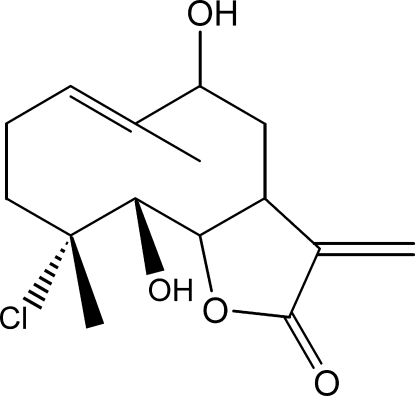


## Experimental

### 

#### Crystal data

C_15_H_21_ClO_4_
*M* *_r_* = 300.77
Orthorhombic, 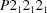

*a* = 8.0224 (2) Å
*b* = 12.1532 (2) Å
*c* = 15.4147 (4) Å
*V* = 1502.90 (6) Å^3^
*Z* = 4
Mo *K*α radiation
μ = 0.26 mm^−1^
*T* = 180 K
0.35 × 0.27 × 0.17 mm

#### Data collection

Agilent Xcalibur Eos Gemini ultra diffractometer
Absorption correction: multi-scan (*CrysAlis PRO*; Agilent, 2010 ▶) *T*_min_ = 0.889, *T*_max_ = 1.000
8982 measured reflections
3053 independent reflections
2944 reflections with *I* > 2σ(*I*)
*R*_int_ = 0.019

#### Refinement

*R*[*F* ^2^ > 2σ(*F* ^2^)] = 0.027
*wR*(*F* ^2^) = 0.070
*S* = 1.04
3053 reflections
185 parameters
H-atom parameters constrained
Δρ_max_ = 0.21 e Å^−3^
Δρ_min_ = −0.21 e Å^−3^
Absolute structure: Flack (1983 ▶), 614 Friedel pairs
Flack parameter: −0.06 (5)

### 

Data collection: *CrysAlis PRO* (Agilent, 2010 ▶); cell refinement: *CrysAlis PRO*; data reduction: *CrysAlis PRO*; program(s) used to solve structure: *SHELXS97* (Sheldrick, 2008 ▶); program(s) used to refine structure: *SHELXL97* (Sheldrick, 2008 ▶); molecular graphics: *ORTEP-3 for Windows* (Farrugia, 1997 ▶) and *PLATON* (Spek, 2009 ▶); software used to prepare material for publication: *WinGX* (Farrugia, 1999 ▶).

## Supplementary Material

Crystal structure: contains datablock(s) I, global. DOI: 10.1107/S1600536811039717/im2319sup1.cif

Structure factors: contains datablock(s) I. DOI: 10.1107/S1600536811039717/im2319Isup2.hkl

Supplementary material file. DOI: 10.1107/S1600536811039717/im2319Isup3.cml

Additional supplementary materials:  crystallographic information; 3D view; checkCIF report

## Figures and Tables

**Table 1:** Hydrogen-bond geometry (Å, °)

*D*—H⋯*A*	*D*—H	H⋯*A*	*D*⋯*A*	*D*—H⋯*A*
O3—H3⋯O4^i^	0.82	1.96	2.763 (1)	167
O4—H4⋯O3^ii^	0.82	2.17	2.980 (1)	171

Symmetry codes: (i) 
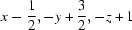
; (ii) 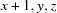.

**Fig. 1.**
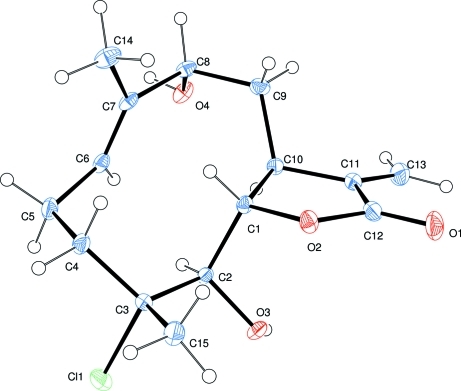
: Molecular structure of the title compound with the atom-labelling scheme. Displacement ellipsoids are drawn at the 30% probability level. H atoms are represented as small spheres of arbitrary radii.

**Fig. 2.**
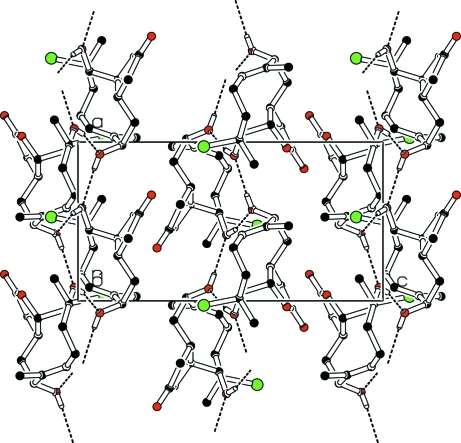
: Packing view showing the C–H···O hydrogen bonds as dashed lines. H atoms not involved in hydrogen bonding have been omitted for clarity.

## supplementary crystallographic information

### Comment

Our work lies within the framework of the evaluation of medicinal plants and in particular, *Anvillea radiata*. The main constituent of the chloroform extract of the aerial parts of *Anvillea radiata* is 9α-hydroxyparthenolide (El Hassany *et al.*, 2004). The reactivity of this sesquiterpene lactone and its derivatives has been the subject of several studies (Castaneda-Acosta *et al.*, 1993; Neukirch *et al.*, 2003; Hwang *et al.*, 2006; Neelakantan *et al.*, 2009), in order to prepare products with a high added value that can be used in the pharmacological industry. In the same context, we have treated 9α-hydroxyparthenolide with 5% of titanium tetrachloride (TiCl_4_)and obtained (1*S*, 2*R*, 3*R*, 8*R*, 10*S*)-3-chloro-2,8- dihydroxy-3,7-dimethyl-11-methylene-13-oxabicyclo[8.3.0]tridec-6-en-12-one with a yield of 52%. The structure of this new product was determined by its single-crystal X-ray structure. The molecule contains two fused rings which exhibit different conformations. The molecular structure of the title compound, Fig.1, shows the lactone ring to adopt an envelope conformation, as indicated by Cremer & Pople (1975) puckering parameters QT = 0.147 (2) Å and φ2 = 58.1 (5)°. The ten-membered ring displays an approximate chair-chair conformation.In the crystal structure, molecules are linked into chains (Fig. 2) running along the *a* axis by intermolecular O—H···O hydrogen bonds (Table 1). Owing to the presence of Cl atom, the absolute configuration could be fully confirmed, by refining the Flack parameter (Flack, 1983) as C1(*S*), C2(*R*), C3(*R*), C8(*R*)and C10(*S*).

### Experimental

To a solution of 9α-hydroxyparthenolide (500 mg, 1.89 mmol) in 20 ml dichloromethane are added in small portions and carefully a catalytic amount (5%) of titanium tetrachloride (TiCl_4_). The reaction mixture was kept at room temperature and stirred for 3 h. Afterwards it was hydrolysed with 20 ml of water and extracted three times with dichloromethane (20 mL). The organic phases are combined, dried over anhydrous Na_2_SO_4_ and then evaporated under reduced pressure. The resulting residue is purified by chromatography on silica gel with hexane /ethyl acetate (30/70) as eluent. This allowed us to isolate in pure 291 mg (0,98 mmol, 52%) of (1*S*, 2*R*, 3*R*, 8*R*, 10*S*)-3-chloro-2,8-dihydroxy-3,7-dimethyl-11-methylene- 13-oxabicyclo[8.3.0]tridec-6-en-12-one. The title compound was recrystallized from ethyl acetate to produce crystals suitable for X-ray diffraction.

### Refinement

All H atoms were fixed geometrically and treated as riding with C—H = 0.96 Å (methyl), 0.97 Å (methylene), 0.98 Å (methine) and O–H = 0.82 Å with *U*_iso_(H) = 1.2*U*_eq_(methylene, methine) or *U*_iso_(H) = 1.5*U*_eq_ (methyl, OH).

### Crystal data

**Table:**

C_15_H_21_ClO_4_	*F*(000) = 640
*M**_r_* = 300.77	*D*_x_ = 1.329 Mg m^−^^3^
Orthorhombic, *P*2_1_2_1_2_1_	Mo *K*α radiation, λ = 0.71073 Å
Hall symbol: P 2ac 2ab	Cell parameters from 8982 reflections
*a* = 8.0224 (2) Å	θ = 3.6–26.4°
*b* = 12.1532 (2) Å	µ = 0.26 mm^−^^1^
*c* = 15.4147 (4) Å	*T* = 180 K
*V* = 1502.90 (6) Å^3^	Prism, colourless
*Z* = 4	0.35 × 0.27 × 0.17 mm

### Data collection

**Table:**

Agilent Xcalibur Eos Gemini ultra diffractometer	3053 independent reflections
Radiation source: Enhance (Mo) X-ray Source	2944 reflections with *I* > 2σ(*I*)
graphite	*R*_int_ = 0.019
Detector resolution: 16.1978 pixels mm^-1^	θ_max_ = 26.4°, θ_min_ = 3.6°
ω scans	*h* = −10→9
Absorption correction: multi-scan (*CrysAlis PRO*; Agilent, 2010)	*k* = −15→15
*T*_min_ = 0.889, *T*_max_ = 1.000	*l* = −18→19
8982 measured reflections	

### Refinement

**Table:**

Refinement on *F*^2^	Secondary atom site location: difference Fourier map
Least-squares matrix: full	Hydrogen site location: inferred from neighbouring sites
*R*[*F*^2^ > 2σ(*F*^2^)] = 0.027	H-atom parameters constrained
*wR*(*F*^2^) = 0.070	*w* = 1/[σ^2^(*F*_o_^2^) + (0.0362*P*)^2^ + 0.3492*P*] where *P* = (*F*_o_^2^ + 2*F*_c_^2^)/3
*S* = 1.04	(Δ/σ)_max_ = 0.001
3053 reflections	Δρ_max_ = 0.21 e Å^−^^3^
185 parameters	Δρ_min_ = −0.21 e Å^−^^3^
0 restraints	Absolute structure: Flack (1983), 614 Friedel pairs
Primary atom site location: structure-invariant direct methods	Flack parameter: −0.06 (5)

### Special details

**Table:**

Experimental. Empirical absorption correction using spherical harmonics, implemented in SCALE3 ABSPACK scaling algorithm. CrysAlisPro (Agilent, 2010)
Geometry. All s.u.'s (except the s.u. in the dihedral angle between two l.s. planes) are estimated using the full covariance matrix. The cell s.u.'s are taken into account individually in the estimation of s.u.'s in distances, angles and torsion angles; correlations between s.u.'s in cell parameters are only used when they are defined by crystal symmetry. An approximate (isotropic) treatment of cell s.u.'s is used for estimating s.u.'s involving l.s. planes.
Refinement. Refinement of *F*^2^ against ALL reflections. The weighted *R*-factor *wR* and goodness of fit *S* are based on *F*^2^, conventional *R*-factors *R* are based on *F*, with *F* set to zero for negative *F*^2^. The threshold expression of *F*^2^ > σ(*F*^2^) is used only for calculating *R*-factors(gt) *etc*. and is not relevant to the choice of reflections for refinement. *R*-factors based on *F*^2^ are statistically about twice as large as those based on *F*, and *R*- factors based on ALL data will be even larger.

### Fractional atomic coordinates and isotropic or equivalent isotropic displacement parameters (Å^2^)

**Table:**

	*x*	*y*	*z*	*U*_iso_*/*U*_eq_	
Cl	0.47204 (6)	0.42068 (3)	0.58743 (3)	0.03475 (11)	
C1	0.60869 (16)	0.59828 (11)	0.36912 (9)	0.0169 (3)	
H1	0.6725	0.5379	0.3432	0.020*	
C2	0.55399 (17)	0.56590 (10)	0.46192 (9)	0.0169 (3)	
H2	0.6465	0.5833	0.5011	0.020*	
C3	0.51598 (18)	0.44248 (11)	0.47165 (9)	0.0193 (3)	
C4	0.66321 (18)	0.36766 (11)	0.44947 (11)	0.0226 (3)	
H4A	0.6774	0.3691	0.3870	0.027*	
H4B	0.6322	0.2931	0.4651	0.027*	
C5	0.83398 (18)	0.39143 (12)	0.49062 (11)	0.0271 (3)	
H5A	0.8208	0.3935	0.5532	0.033*	
H5B	0.9085	0.3310	0.4770	0.033*	
C6	0.91472 (16)	0.49718 (12)	0.46184 (10)	0.0211 (3)	
H6	0.9289	0.5519	0.5034	0.025*	
C7	0.96724 (17)	0.51863 (11)	0.38212 (10)	0.0201 (3)	
C8	1.01946 (18)	0.63373 (12)	0.35599 (10)	0.0213 (3)	
H8	1.1106	0.6281	0.3139	0.026*	
C9	0.87577 (18)	0.69698 (12)	0.31401 (10)	0.0231 (3)	
H9A	0.8484	0.6619	0.2593	0.028*	
H9B	0.9135	0.7711	0.3012	0.028*	
C10	0.71583 (16)	0.70436 (11)	0.36933 (10)	0.0181 (3)	
H10	0.7458	0.7230	0.4292	0.022*	
C11	0.59596 (18)	0.78895 (13)	0.33507 (10)	0.0228 (3)	
C12	0.44945 (19)	0.73049 (13)	0.29884 (11)	0.0264 (3)	
C13	0.6075 (2)	0.89766 (13)	0.33538 (12)	0.0329 (4)	
H13A	0.5217	0.9401	0.3123	0.039*	
H13B	0.7013	0.9316	0.3587	0.039*	
C14	0.9722 (2)	0.43627 (13)	0.30957 (11)	0.0296 (3)	
H14A	0.9579	0.3636	0.3327	0.044*	
H14B	1.0777	0.4409	0.2804	0.044*	
H14C	0.8842	0.4517	0.2692	0.044*	
C15	0.36070 (18)	0.40750 (12)	0.42250 (11)	0.0267 (3)	
H15A	0.3759	0.4215	0.3617	0.040*	
H15B	0.2666	0.4485	0.4433	0.040*	
H15C	0.3415	0.3304	0.4314	0.040*	
O1	0.33335 (15)	0.76689 (11)	0.25906 (9)	0.0408 (3)	
O2	0.46321 (13)	0.62157 (8)	0.31574 (7)	0.0235 (2)	
O3	0.41528 (12)	0.63183 (8)	0.48730 (7)	0.0219 (2)	
H3	0.4483	0.6844	0.5159	0.033*	
O4	1.07598 (12)	0.69787 (8)	0.42835 (8)	0.0261 (2)	
H4	1.1646	0.6730	0.4461	0.039*	

### Atomic displacement parameters (Å^2^)

**Table:**

	*U*^11^	*U*^22^	*U*^33^	*U*^12^	*U*^13^	*U*^23^
Cl	0.0427 (2)	0.03388 (19)	0.0276 (2)	0.00420 (18)	0.00966 (18)	0.00940 (16)
C1	0.0124 (6)	0.0175 (6)	0.0206 (7)	0.0001 (5)	−0.0032 (5)	0.0002 (5)
C2	0.0130 (6)	0.0169 (6)	0.0208 (7)	0.0019 (5)	0.0001 (5)	−0.0004 (5)
C3	0.0188 (7)	0.0195 (6)	0.0195 (7)	0.0008 (5)	0.0027 (6)	0.0042 (5)
C4	0.0204 (7)	0.0152 (6)	0.0322 (8)	0.0012 (5)	0.0019 (6)	0.0009 (6)
C5	0.0201 (7)	0.0237 (8)	0.0374 (9)	0.0051 (6)	−0.0026 (7)	0.0084 (6)
C6	0.0134 (6)	0.0208 (6)	0.0290 (8)	0.0032 (5)	−0.0038 (6)	−0.0013 (6)
C7	0.0109 (6)	0.0205 (6)	0.0289 (8)	0.0014 (5)	−0.0007 (6)	−0.0065 (6)
C8	0.0150 (7)	0.0231 (7)	0.0257 (7)	−0.0018 (5)	0.0029 (6)	−0.0057 (6)
C9	0.0200 (7)	0.0253 (7)	0.0240 (8)	−0.0045 (6)	0.0034 (6)	0.0022 (6)
C10	0.0155 (6)	0.0179 (6)	0.0208 (7)	−0.0011 (5)	−0.0002 (6)	0.0018 (6)
C11	0.0201 (7)	0.0255 (7)	0.0228 (8)	0.0007 (6)	0.0026 (6)	0.0061 (6)
C12	0.0217 (7)	0.0308 (8)	0.0267 (8)	−0.0005 (6)	0.0007 (7)	0.0100 (6)
C13	0.0331 (9)	0.0247 (8)	0.0408 (10)	0.0031 (7)	0.0023 (8)	0.0073 (7)
C14	0.0240 (8)	0.0288 (8)	0.0361 (9)	−0.0018 (6)	0.0042 (7)	−0.0135 (7)
C15	0.0191 (7)	0.0201 (7)	0.0410 (9)	−0.0039 (6)	−0.0041 (7)	0.0039 (7)
O1	0.0255 (6)	0.0461 (7)	0.0507 (8)	0.0016 (5)	−0.0121 (6)	0.0243 (6)
O2	0.0193 (5)	0.0256 (5)	0.0255 (5)	−0.0039 (4)	−0.0078 (5)	0.0052 (4)
O3	0.0156 (5)	0.0193 (5)	0.0308 (6)	0.0022 (4)	0.0021 (4)	−0.0050 (4)
O4	0.0169 (5)	0.0233 (5)	0.0380 (7)	0.0005 (4)	−0.0052 (4)	−0.0100 (5)

### Geometric parameters (Å, °)

**Table:**

Cl—C3	1.8384 (15)	C8—C9	1.529 (2)
C1—O2	1.4557 (16)	C8—H8	0.9800
C1—C2	1.5471 (19)	C9—C10	1.5433 (19)
C1—C10	1.5495 (18)	C9—H9A	0.9700
C1—H1	0.9800	C9—H9B	0.9700
C2—O3	1.4260 (16)	C10—C11	1.504 (2)
C2—C3	1.5380 (18)	C10—H10	0.9800
C2—H2	0.9800	C11—C13	1.324 (2)
C3—C15	1.519 (2)	C11—C12	1.483 (2)
C3—C4	1.5292 (19)	C12—O1	1.1997 (19)
C4—C5	1.537 (2)	C12—O2	1.3536 (18)
C4—H4A	0.9700	C13—H13A	0.9300
C4—H4B	0.9700	C13—H13B	0.9300
C5—C6	1.506 (2)	C14—H14A	0.9600
C5—H5A	0.9700	C14—H14B	0.9600
C5—H5B	0.9700	C14—H14C	0.9600
C6—C7	1.325 (2)	C15—H15A	0.9600
C6—H6	0.9300	C15—H15B	0.9600
C7—C14	1.5014 (19)	C15—H15C	0.9600
C7—C8	1.5148 (19)	O3—H3	0.8200
C8—O4	1.4343 (17)	O4—H4	0.8200
			
O2—C1—C2	110.16 (11)	O4—C8—H8	108.5
O2—C1—C10	106.51 (10)	C7—C8—H8	108.5
C2—C1—C10	111.53 (11)	C9—C8—H8	108.5
O2—C1—H1	109.5	C8—C9—C10	114.96 (12)
C2—C1—H1	109.5	C8—C9—H9A	108.5
C10—C1—H1	109.5	C10—C9—H9A	108.5
O3—C2—C3	111.50 (11)	C8—C9—H9B	108.5
O3—C2—C1	109.40 (11)	C10—C9—H9B	108.5
C3—C2—C1	113.23 (11)	H9A—C9—H9B	107.5
O3—C2—H2	107.5	C11—C10—C9	112.17 (12)
C3—C2—H2	107.5	C11—C10—C1	102.32 (11)
C1—C2—H2	107.5	C9—C10—C1	114.31 (12)
C15—C3—C4	110.83 (12)	C11—C10—H10	109.3
C15—C3—C2	112.77 (11)	C9—C10—H10	109.3
C4—C3—C2	113.89 (11)	C1—C10—H10	109.3
C15—C3—Cl	106.66 (10)	C13—C11—C12	122.32 (15)
C4—C3—Cl	106.23 (10)	C13—C11—C10	129.51 (15)
C2—C3—Cl	105.86 (9)	C12—C11—C10	108.16 (13)
C3—C4—C5	118.97 (12)	O1—C12—O2	121.48 (15)
C3—C4—H4A	107.6	O1—C12—C11	129.15 (15)
C5—C4—H4A	107.6	O2—C12—C11	109.36 (13)
C3—C4—H4B	107.6	C11—C13—H13A	120.0
C5—C4—H4B	107.6	C11—C13—H13B	120.0
H4A—C4—H4B	107.0	H13A—C13—H13B	120.0
C6—C5—C4	114.97 (12)	C7—C14—H14A	109.5
C6—C5—H5A	108.5	C7—C14—H14B	109.5
C4—C5—H5A	108.5	H14A—C14—H14B	109.5
C6—C5—H5B	108.5	C7—C14—H14C	109.5
C4—C5—H5B	108.5	H14A—C14—H14C	109.5
H5A—C5—H5B	107.5	H14B—C14—H14C	109.5
C7—C6—C5	125.30 (14)	C3—C15—H15A	109.5
C7—C6—H6	117.3	C3—C15—H15B	109.5
C5—C6—H6	117.3	H15A—C15—H15B	109.5
C6—C7—C14	124.62 (13)	C3—C15—H15C	109.5
C6—C7—C8	121.08 (13)	H15A—C15—H15C	109.5
C14—C7—C8	114.22 (12)	H15B—C15—H15C	109.5
O4—C8—C7	112.49 (12)	C12—O2—C1	111.36 (11)
O4—C8—C9	107.12 (11)	C2—O3—H3	109.5
C7—C8—C9	111.61 (12)	C8—O4—H4	109.5

### Hydrogen-bond geometry (Å, °)

**Table:**

*D*—H···*A*	*D*—H	H···*A*	*D*···*A*	*D*—H···*A*
O3—H3···O4^i^	0.82	1.96	2.763 (1)	167
O4—H4···O3^ii^	0.82	2.17	2.980 (1)	171

Symmetry codes: (i) *x*−1/2, −*y*+3/2, −*z*+1; (ii) *x*+1, *y*, *z*.
